# Interventions to increase use of services; Mental Health Awareness in Nigeria

**DOI:** 10.1186/s13033-017-0173-z

**Published:** 2017-10-24

**Authors:** Julian Eaton, Emeka Nwefoh, Godwin Okafor, Ugochukwu Onyeonoro, Kenneth Nwaubani, Claire Henderson

**Affiliations:** 1 CBM International and London School of Hygiene and Tropical Medicine, London, UK; 2 CBM Country Co-ordination Office, Abuja, Nigeria; 3 Federal Medical Centre, Umuahia, Abia State Nigeria; 4 Amaudo Itumbauzo, Bende, Abia State Nigeria; 5 King’s College Institute of Psychiatry, Psychology and Neurosciences, London, UK

**Keywords:** Community Mental Health, Village Health Workers, Psychiatry, Mental Health Awareness, Programme, Nigeria, Primary care, Help seeking behaviour

## Abstract

**Background:**

Mental health services in Nigeria consist mainly of large government psychiatric hospitals and there are very few mental health professionals to serve the large population of the country. However, more recently, community mental health services, which have been shown to improve access to care and clinical outcomes are beginning to develop in some locations. Despite efforts to promote more accessible services, low levels of knowledge about effective treatment of mental disorders means that even where these services are available, a very small proportion of people utilise these services. Therefore interventions to increase service use are an essential component of health system.

**Methods:**

This intervention was designed to increase use of a mental health services through the work of community-based Village Health Workers. Fifteen Village Health Workers in each Local Government Area (district) were selected and trained to create mental health awareness in communities. Their function also include identification and referral of persons with mental illness to trained mental health nurses in the clinics. Attendance data prior to and after intervention were collected and compared.

**Results:**

The incident rate for initial period of intervention is five times higher than the baseline rate (95% CI; 3.42–7.56; p < 0.001) though this diminished in the long term, levelling off above initial baseline.

**Conclusions:**

This study demonstrated that addition of awareness raising using volunteers in communities as part of health programme implementation can increase services use by a population. Mechanisms such as informing populations of the existence of a service which they were previously lacking; explanation of causation of mental illness and achieving community leaders’ support for a new service can make investment in services more efficient by increasing attendance.

## Background—services for people with mental illness

In Nigeria less than 15% of people with severe mental illness access mental health care services [1]. As with other countries in sub-Saharan Africa, mental health care is neglected, and neuropsychiatric services receive low priority in national budget allocations [2], with only around 1% of the health budget spent on mental health [3], whereas the proportion of the burden of disease attributable to mental illness is around 8% in the same region [4]. These funds are also spent inefficiently; mental health services in Nigeria consist mainly of large government psychiatric hospitals. There are eight Federal Neuro-Psychiatric Hospitals and a similar number of university teaching hospital psychiatric departments, for a population of 170 million people. Nigeria has around one psychiatrist per 1 million population and four psychiatric nurses per 100,000 people [5]. However, the country is starting to develop community mental health services, which have been shown to improve access to care and clinical outcomes [6–8]. Theoretical models related to stigma imply that reduction in florid symptoms, that lead others to label a person as having a mental illness and hence stereotype them as being unpredictable and dangerous, would reduce their experience of stigma and discrimination [9]. Despite recent efforts to promote more accessible services, low levels of knowledge about effective treatment of mental disorders means that even where it is available, a very small proportion of people receive appropriate care [10]. Interventions to increase service use are therefore an essential component of the health systems approach to reducing the treatment gap for mental illness.

Effective techniques to improve knowledge about, and attitudes towards, people with mental illness include educating key influential groups and those with frequent contact with people with mental illness [11, 12]. Most work to date has been concentrated on experience in high income settings, but there are increasing examples in low income setting of interventions to address knowledge and stigma associated with mental illness [13, 14], In addition, studies on combating the stigma associated with leprosy and HIV/AIDS suggest that interventions based in such settings can be effective [15, 16].

A previous programme in this part of Nigeria found that a community awareness programme resulted in significantly increased clinic attendance [17]. In this programme, community-based volunteers underwent a week long course to gain a basic understanding of mental health, and training in which they were taught to share key messages, and identify and refer people in the community with mental health problems. The intervention resulted in a significant increase in referral rates. This study aimed to replicate this work, and add rigour by including a comparison site in the study design. Our primary hypothesis was that; compared to the control State, the intervention State would show a significant increase in presentation rates to Community Mental Health Programme (CMHP) clinics.

## Study design and methods

### The Mental Health Awareness Programme intervention

The Mental Health Awareness Programme (MHAP) was an initiative of a local Non-Governmental Organisation (NGO) in South East Nigeria called Amaudo (or Village of Peace in the local Igbo language). Amaudo was initially established in 1990 to support homeless people with severe mental illness, and the Community Mental Health Programme was added to this work 10 years later [18]. The services are provided as a partnership with the State and Local Governments in South-East Nigeria. Nigeria’s health system reflects its Federal structure of governance, with specialist tertiary services being largely the mandate of Federal Government, each of the 36 States running secondary hospital services, and Local Government Areas (LGAs) running primary care. Local Government Areas are a legislative level that may be considered equivalent to a large health district, under which there are usually many primary care centres of different sizes, organised under health wards. Under CMHP then, LGAs were identified as the unit under which the services would be organised. Services are run by a psychiatric nurse placed in one primary care clinic—usually the largest—in each LGA in the State. This nurse provides outpatient services, and also makes domiciliary visits and acts as a point of referral for other health workers. The awareness programme was specifically designed to strengthen this service and used the network of CMHP clinics (and psychiatric nurses) in its three States of operation in Nigeria (Abia, Imo and Ebonyi States) as well as in Anambra and Enugu States where the CMHP planned to expand. Its aims were to increase awareness about human rights of people with mental illness (and epilepsy); to change attitudes and reduce discrimination; and to increase the number of people using primary health care for mental/neurological illness, in line with the Nigerian National Policy for Mental Health [19].

The MHAP intervention was designed to increase use of a mental health service in Nigeria, by utilising Village Health Workers (VHWs), who work in communities, usually in a health promotion role such as vaccination programmes or HIV/AIDS awareness. As local volunteers linked to health services, they have a unique engagement with members of communities, including people with mental health conditions, and their carers [20]. 15 VHWs in each Local Government Area were identified using clear criteria designed to engage those who were motivated to work in mental health, and were likely to remain in the community and role for some time. A total of 315 VHW were identified and each group of 15 received a 5 day training by the psychiatric nurse in their local clinic, using a package that had been previously developed and tested by Amaudo. The content focused on gaining knowledge about mental illness, promoting human rights and dignity, and sharing practical ways of supporting social integration. The fact that the nurse provided the training reinforced relationships for ongoing work between the nurse and the VHWs. In addition, key local actors such as Primary Health Care Coordinators and other local health leaders were invited to supervise the training, and local community leaders participated in opening ceremonies. Further details are available from http://www.amaudouk.org. Following the training, they were then expected to visit each village in their catchment area, share information using the provided materials, and encourage people identified as having mental health problems to attend the clinic. The awareness-raising activities in communities involved speaking to community leaders, at community gatherings (including women’s and youth meetings), and in church meetings. At the same time, a media campaign was run involving brief messages on the radio, and use of posters and leaflets. VHWs received a 1 day refresher training 6 months after the initial training, and maintained a relationship with the psychiatric nurse. This intervention has been described previously in previous published work [17] and a programme report [21].

The study used the phased roll-out of the programme to measure impact during the MHAP implementation in Imo State (intervention), and used Anambra States as a control during the year prior to implementation there. We selected Imo as the intervention state (population 3.9 million, 21 active clinics) and Anambra State (population 4.2 million, 21 clinics) as the control because they have similar demography and health systems, and the CMHP services were identical, including VHW presence.

Routinely available attendance data from all clinics in the service in the intervention and control state were collected (42 in total). Unfortunately, there was a nurse strike in our control state (Anambra) during the period where we needed to collect service use data, so we were unable to compare concurrent data using comparison of mean attendance with the t test. We adjusted the analysis instead to rely on a comparison with data from Imo prior to the intervention.

## Results

### Change in number of new patients using clinics

The statistical analysis had to be adjusted to take account of the lack of a control site. The negative binomial model used is an extension to the Poisson model for analyzing count data in that it allows for the over-dispersion of zeros that would be uncharacteristic of the more familiar Poisson distribution. The results however can be interpreted in the normal fashion in the form of incident rate ratios (IRR). In the context of the model above, IRRs higher than 1 indicate an increased risk of the event occurring (i.e. higher number of new patients) and IRRs lower than 1 indicates a lower rate of new patients.

The model is adjusted for an underlying baseline risk that has been tested for non-linearity but appears to be linear in nature (i.e. constant). With increasing months, the risk of increased number of new patients is 1.000 indicating that the risk does not change with time (i.e. the gradient of the slope is 0, flat). Thus there was no significant trend for the number of new patients seen from beginning of the data collection up to and including June 2011.

The incident rate for the initial period of the intervention was 5.1 times higher than the incident rate for the baseline rate (which we have shown to be a flat trend) and this increase was highly statistically significant (95% CI 3.42–7.56, p < 0.001).

### The long-term effect of the intervention

**Fig. 1:**
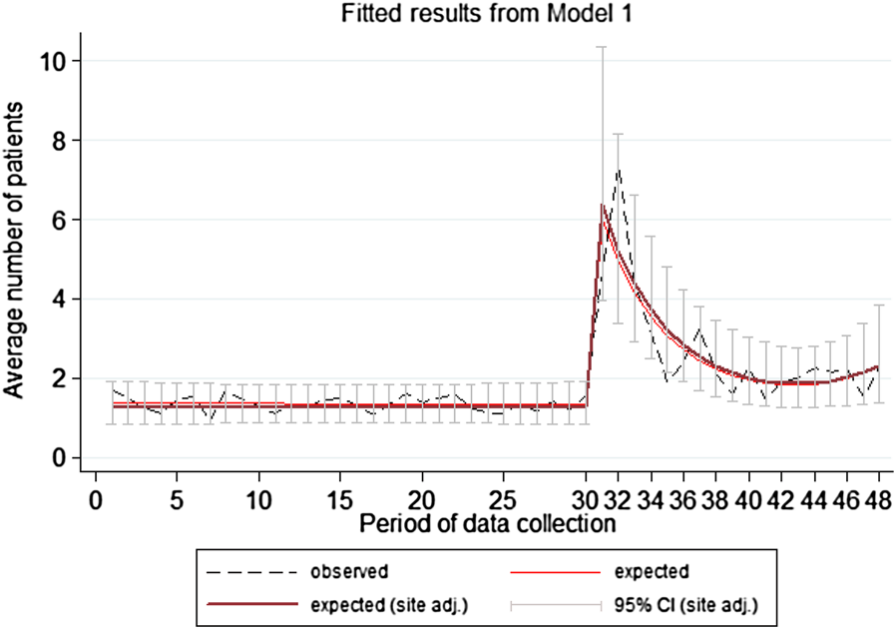
Fitted regression estimates for the incidence of new patients per month

This initial significant effect quickly diminished, and the intervention effect reduced before leveling off above the initial baseline (see Fig. 1).

**Fig. 2:**
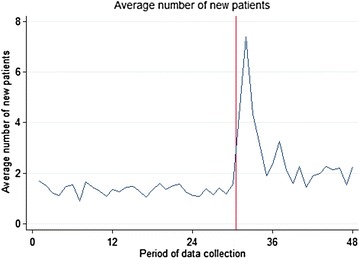
Mean number of new patients seen per month

Figure 1 shows the expected results calculated from the first model above. The fitted regression line (maroon) closely fits the observed data (black dash line) that was seen previously in Fig. 2, and closely describes the change in the number of new patients seen each month. The red line shows fitted results unadjusted by site for reference.

This shape of curve is seen frequently following an intervention whereby the initial effect is not sustained and eventually levels at a smaller effect or returns to the baseline. Unfortunately we do not have enough points of data collection after the intervention to estimate the stable intervention effect as the rate of new patients seen each month may or may not be changing by the end of data collection. Also, local fluctuations towards the end of the data collection appear slightly increased, but the figures fall within the range of fluctuations seen in the data.

Another way of describing the intervention effect is to use this regression equation to see at which points following the intervention is the rate of new patients seen higher than the baseline rate. See Table 1.

**Table 1:** Treatment effect with time

Treatment effect with time	IRR	95% CI (LL–UL)	p value
0—initial intervention effect (July 2011)	5.09	(3.42–7.56)	< 0.001
1st period after the intervention (August 2011)	4.18	(2.94–5.93)	< 0.001
2nd period after the intervention (September 2011)	3.49	(2.52–4.84)	< 0.001
3rd (October 2011)	2.97	(2.15–4.09)	< 0.001
4th (November 2011)	2.56	(1.84–3.56)	< 0.001
5th (December 2011)	2.25	(1.60–3.17)	< 0.001
6th (January (2012)	2.02	(1.41–2.89)	< 0.001
7th (February 2012)	1.83	(1.26–2.66)	0.001
8th (March 2012)	1.70	(1.15–2.50)	0.007
9th (April 2012)	1.60	(1.08–2.38)	0.020
10th (May 2012)	1.53	(1.02–2.30)	0.038
11th (June 2012)	1.49	(0.99–2.25)	0.056
12th (July 2012)	1.48	(0.97–2.25)	0.066
13th (August 2012)	1.49	(0.97–2.30)	0.067
14th (September 2012)	1.53	(0.98–2.40)	0.062
15th (October 2012)	1.60	(0.99–2.58)	0.053
16th (November 2012)	1.70	(1.01–2.85)	0.044
17th (December 2012)	1.84	(1.04–3.27)	0.037

Table 1 compares the intervention effect at each time-point and compares it to the baseline rate of new patients seen per month. As described previously, the rate of new patients seen in the initial intervention was 5.09 times higher than the baseline. By the following month, the number of new patients seen per month was 4.18 times higher than the baseline (p < 0.001). Up to and including the 10th month following the intervention, the number of new patients seen per month was higher than the baseline rate. Following this, the rate of new patients seen each month remains above baseline until the end of data collection, but the results are not statistically significant for a period, before tending in this direction again.

## Discussion

Carrying out implementation of innovative programmes is challenging in Nigeria, particularly in the public sector. This is in part due to the fact that systems of management and bureaucracy were not very efficient, and due to the frequency of disruptions in services. Despite the fact that political circumstances resulted in a change of available data, which required a different statistical analysis, we have been able to demonstrate that an awareness intervention can significantly increase service use (in this case a fivefold increase). These results show that addition of an awareness raising component, delivered by lay community workers, as part of health programme implementation can increase use of services by the target population.

Potential mechanisms for the increase in service use observed include simply informing populations of the existence of a service of which they were previously unaware (but that they would be inclined to use), to impacting on explanatory models of causation of mental illness (and challenging negative ideas of prognosis for example), or the effect of community leaders’ endorsement of a new service.

The initial increase in service use may well have been in part due to large numbers of people making use of the service on discovering its availability (it was not a new service). The sharp reduction in new patients is not entirely justified by this, however, because the treatment gap far exceeds even the increased numbers accessing the service. The awareness intervention covered both common mental disorders, severe mental illness (especially psychosis) and epilepsy. It is worth noting that a high proportion of service users had epilepsy and severe mental illness, compared to local population prevalence. This is a common finding, representing pathways to care, identification of common mental disorders, and somatization issues.

Even interventions that are deliberately designed to penetrate the most remote communities will always first reach those who are closest to making use of the service, because they are able to reach it, to afford it, or would tend to use formal health services for mental health needs. Alternatively, those who have already used traditional options without satisfaction may be ready to try a new option available to them. Further qualitative research would shed light on which of these processes are relevant.

The up-turn in attendance at the end of data collection may have been due to the refresher training carried out on average 6 months after the initial training, though a longer data collection period would have made this clearer. A greater sustained effect might have been increased by more intense use (increasing the dose) of active elements of the intervention like VHW refresher training (which has a motivating effect in addition to reinforcing knowledge). In addition, other elements of a comprehensive intervention, like media campaigns, are recognised to contribute to the overall effect [22].

The lack of a control site was an important limitation in the study. While we can reasonably attribute the change to the intervention, this would have been more appropriately tested with the control group as originally planned. In addition, while an increase in service use was demonstrated, it is not possible to know what element of the intervention was responsible for this effect, as the study did not explore this a methodology that allowed such analysis.

## Conclusions

Our results imply that targeting key community actors is an effective way of improving help-seeking in areas where formal health services are not necessarily seen within local belief systems as the obvious place to seek treatment for mental illness. Further research might explore potential mechanisms for change in attendance and service use behaviour using qualitative methods.

In this case, although the existing health intervention (a community mental health programme) did not change, there was a significant increase in use of the service following an awareness-raising intervention using village-based health workers. This was sustained for almost a year, but there was a clear reduction in the effect with time. This might be addressed by ensuring ongoing engagement and repetition of active ingredients of the intervention.

## Abbreviations

CBM: formerly Christoffel Blindenmission
CMHP: Community Mental Health Programme
HIV/AIDS: Human Immunodeficiency Virus and Acquired Immunodeficiency Diseases
IRR: incident rate ratios
LAMICs: low and middle income countries
MHAP: Mental Health Awareness Programme
NGO: Non-Governmental Organisation
PHC: Primary Health Centre
VHWs: Village Health Workers
